# Two TPL-Binding Motifs of ARF2 Are Involved in Repression of Auxin Responses

**DOI:** 10.3389/fpls.2018.00372

**Published:** 2018-03-21

**Authors:** Hee-Seung Choi, Minji Seo, Hyung-Taeg Cho

**Affiliations:** Department of Biological Sciences, Seoul National University, Seoul, South Korea

**Keywords:** auxin, auxin signaling, EAR motif, repressor auxin response factor (rARF), TPL/TPR-binding motif

## Abstract

Auxin signaling is finalized by activator auxin response factors (aARFs) that are released from Auxin/Indole-3-Acetic Acid (Aux/IAA) repressors and directly activate auxin-responsive genes. However, it remains to be answered how repressor ARFs (rARFs) exert their repression function. In this study, we assessed the molecular and biological functions of two putative co-repressor-binding motifs (EAR and RLFGI) of ARF2 (a rARF) in *Arabidopsis thaliana*. In the yeast two-hybrid assay, the EAR mutation moderately and the RLFGI mutation, or both motifs, almost completely disrupted the interaction between the co-repressor TOPLESS (TPL) and the repressive motifs-containing middle domain (MD) of ARF2. The ARF2-MD interacted not only with TPL but also with TPL homologs (TPRs). Root hair-specific overexpression of rARFs (ARF1–4, 9–11, and 16) considerably inhibited root hair growth, suggesting that rARFs generally function as repressors in the auxin-responsive root hair single cell. Individual mutation of the ARF2 EAR or RLFGI motif slightly and both mutations greatly compromised ARF2-mediated inhibition of root hair growth and auxin-responsive gene expression. In addition, flowering time and seed size, two representative *arf2* mutant phenotypes, were examined to assess the function of the repressive motifs in mutant-complementation experiments. ARF2-mediated inhibition of flowering and seed growth was suppressed considerably by the individual mutation of EAR or RLFGI and almost completely by both mutations. These results suggest that EAR and RLFGI work together as major repressive motifs for ARF2 to recruit TPL/TPR co-repressors and to exhibit its repressive biological functions.

## Introduction

Auxin is an essential hormone for diverse growth and developmental processes in plants. In contrast to its versatile biological functions, the auxin signaling pathway has turned out to be seemingly simple. Three major signaling components have been implicated in auxin signaling; TRANSPORT INHIBITOR RESPONSE1/AUXIN F-BOX (TIR1/AFB) proteins, Auxin/Indole-3-Acetic Acid (Aux/IAA) transcriptional co-regulators, and DNA binding proteins called auxin response factors (ARFs), which have 6, 29, and 23 members in Arabidopsis, respectively (reviewed in Mockatis and Estelle, 2008). The genes necessary for auxin responses are directly regulated by ARFs, and Aux/IAAs function as transcriptional repressors by recruiting the co-repressors TOPLESS (TPL)/TPL-RELATEDs (TPRs) to ARFs. Auxin is perceived by the co-receptors, TIR1/AFBs and Aux/IAAs, and its binding promotes ubiquitination of Aux/IAAs by the E3 ligase complex where TIR1/AFBs act as F-box proteins. Ubiquitinated Aux/IAAs are degraded by the proteasome complex, and free ARFs promote the transcription of auxin responsive genes.

Degradation of Aux/IAAs is the central event in auxin signaling. Most Aux/IAAs consist of three major domains. The N-terminal domain I includes the TPL/TPR-interacting motif, the ETHYLENE RESPONSE FACTOR (ERF)-associated Amphiphilic Repression (EAR) motif, with a LxLxL (L for Leu and x for the particular amino acid) signature (Szemenyei et al., 2008; Kagale et al., 2010). Domain II, a degron, interacts with TIR1/AFBs and auxin. The C-terminal domains III and IV form a Phox and Bem1p (PB1) protein–protein interaction domain (Guilfoyle and Hagen, 2012). The PB1 domain in Aux/IAAs interacts not only with the same domain in Aux/IAAs to multimerize themselves but also with the similar domain in ARFs to recruit TPL/TPRs to and suppress auxin-responsive genes (Ulmasov et al., 1997a,b).

Auxin response factors, DNA-binding transcription factors, bind to the auxin response *cis*-element (AuxRE) via its N-terminal domain I (Ulmasov et al., 1997a). The ARF domain II (middle domain, MD) represents either transcriptional activation or repression activity depending on the ARF member (Ulmasov et al., 1999; Tiwari et al., 2003). The C-terminal PB1 domain in ARFs is similar to that mentioned for Aux/IAAs and is the target of Aux/IAAs for heterodimerization (Kim et al., 1997; Ulmasov et al., 1997a; Tiwari et al., 2003; Korasick et al., 2014). ARFs are classified as activator ARFs (aARFs) or repressor ARFs (rARFs) depending on their transcriptional activity and the molecular structure of their MD, where Gln is rich in aARFs and Ser/Pro/Leu are rich in rARFs (Supplementary Figure S1; Ulmasov et al., 1999; Guilfoyle and Hagen, 2001; Tiwari et al., 2003). The mechanism of how aARFs modulate the transcription of auxin-responsive genes has been well characterized, and is considered equivalent to the auxin signaling pathway. As mentioned above, removal of Aux/IAAs from aARFs in the presence of auxin results in activation of auxin-responsive genes. Conversely, it remains to be answered how rARFs exhibit transcriptional repression activity.

Based on the molecular structure of rARFs, several possibilities can be proposed for the mechanism of rARF-mediated repression of gene expression and auxin responses. First, rARFs may compete with aARFs for binding to AuxRE. Second, rARFs can interact with Aux/IAAs via their PB1 domains and recruit TPL/TPR co-repressors to the AuxRE-including target gene. In this regard, however, a binding assay in yeast cells showed that rARFs scarcely or weakly interact with Aux/IAAs (Vernoux et al., 2011). The less likely interaction between rARFs and Aux/IAAs leads to the other possibility that rARFs may directly recruit TPL/TPRs by using their own TPL/TPR-interacting motif. Many rARFs include putative co-repressor-binding motifs such as LxLxL (EAR) and R/K-LFG-V/I/F (RLFGV) motifs (Supplementary Figures S1, S2; Lokerse and Weijers, 2009).

The EAR motifs of Aux/IAAs, IAA12 (Szemenyei et al., 2008) and IAA7 (Lee et al., 2016), and the RLFGV motif of RAV1 (Causier et al., 2012a) were shown to be necessary for the interaction with TPL. However, it has yet to be demonstrated whether rARF EAR and RLFGV motifs are involved in the interaction with TPL/TPRs and rARF-mediated biological processes. In this study, we chose ARF2, which has both EAR and RLFGV motifs (Supplementary Figure S1), as a model rARF and tested the functions of these two motifs in the interaction with TPL/TPRs and ARF2-mediated developmental processes in Arabidopsis.

## Materials and Methods

### Plant Material and Growth Conditions

*Arabidopsis thaliana*, Columbia ecotype (Col-0), was used as a control and for transformation of transgene constructs unless otherwise stated. Arabidopsis plants were transformed using *Agrobacterium tumefaciens* strain C58C1 (pMP90) by the inflorescence-dipping method. Transformed plants were selected on hygromycin-containing plates (30 μg ml^-1^). All seeds were grown on agarose plates containing 4.3 g ml^-1^ Murashige and Skoog (MS) nutrient mix (Duchefa, Netherlands), 1% sucrose, 0.5 g ml^-1^ MES pH 5.7 with KOH, and 0.8% agarose. Seeds were cold treated before germination at 23°C under a 16 h/8 h light/dark photoperiod. For observation of root hairs, homozygous transformants were planted on antibiotic-free media, and T_1_ and T_2_ lines were planted on hygromycin-containing media. Hygromycin did not significantly interfere with root hair development, as shown in the control *ProE7:YFP* (yellow fluorescence protein) transformants. Two control lines were adopted; WT for the *arf2-6* and *arf2-7* mutant analysis and *ProE7:YFP* (Lee and Cho, 2006; Ganguly et al., 2010) for the transformant analysis with hygromycin. Two *arf2* mutants were obtained from the Arabidopsis Biological Resource Center.

### Construction of Transgenes

To root hair-specifically overexpress ARFs using the root hair-specific *EXPA7* promoter (*ProE7*) for the *ProE7:ARF*s constructs, the *ARF*s sequences were obtained by PCR using Arabidopsis genomic DNA as the template and the primer sets listed in Supplementary Table S1. To generate root hair-specific overexpression lines for wild-type and mutant forms of *ARF2* (genomic wild-type *ARF2*, ma, mb, and mab), sequences were obtained by PCR using the primers listed in Supplementary Table S1. The modified binary vector, *pCAMBIA 1300-NOS*, including *ProE7*, was used as the cloning vector to direct root hair-specific expression and to fuse with *ARF*s and mutant forms of *ARF2*. For the *arf2* mutant complementation experiments, *ProE7* was replaced by *ProARF2* in the *ProE7*-driven constructs for wild-type or mutant ARF2.

For the yeast two-hybrid (Y2H) constructs, cDNAs for full-length *TPL*/*TPR*s, full length *ARF2*, and wild-type and mutant forms of *ARF2* domains were PCR-amplified using the primer sets in Supplementary Table S1 and cloned into the binding domain (BD)-expressing *pGBKT7* or the activation domain (AD)-expressing *pGADT7* vector depending on the experimental design.

All constructs were confirmed by nucleotide sequencing. For Arabidopsis transformation, the *Agrobacterium*-mediated floral dipping method was adopted. Transgene insertion in the Arabidopsis transformants was confirmed by PCR analysis using transgene-specific primers.

### Observation of Biological Parameters

Root hair length was estimated as described in Lee and Cho (2006, 2009) with modifications. The 3-day-old seedling root was digitally photographed using a stereomicroscope (M205 FA, Leica, Heerbrugg, Switzerland) at 40× magnification. The lengths of 9 consecutive hairs protruding perpendicularly from each side of the root, 18 hairs in total, were estimated using ImageJ 1.50b software (National Institutes of Health, United States). For the flowering time analysis, the emergence of a 1-cm-long inflorescence was considered as the bolting time. To estimate seed size, the seeds from control, mutants, or independent T1 transformants were harvested, dried for 2 weeks, digitally photographed under a stereomicroscope, and the seed area calculated using ImageJ 1.50b software.

### RNA Isolation and Quantitative Reverse Transcriptase (qRT)-PCR Analysis

Total RNA was isolated from the roots of 4-day-old seedlings (25 for each line) using an RNeasy Plant Mini Kit (Qiagen). cDNA was synthesized as described previously (Lee and Cho, 2006). qRT-PCR analyses were performed using an amfiSure qGreen Q-PCR Master mix without ROX (Applied GenDEOT) and a Chromo4^TM^ Four-Color Real-Time Detector (Bio-Rad). Gene-specific signals were normalized by the *ACTIN7* transcript level. qRT-PCRs were performed in three technical replications per RNA sample with three independent RNA preparations. Primers used for quantitation were as in Supplementary Table S1.

### Yeast Two-Hybrid Assays

Yeast two-hybrid experiments were performed using the Matchmaker Yeast Two-Hybrid System (Clontech, Palo Alto, CA, United States) with the yeast strain AH109 following the manufacturer’s protocol. Direct interaction of two proteins was investigated by co-transformation of the plasmids into the yeast cell. Transformed cells were cultured in a SD^2-^ medium (lacking Leu and Trp) at 30°C for 3 days. Single colonies were suspended in the SD^2-^ medium, and serial 1:10 dilutions were plated in either SD^2-^, SD^3-^ (lacking Leu, Trp and His), or SD^4-^ (lacking Leu, Trp, His and Ade). 3-amino-triazole (0.2–0.5 mM) was included for cultivation in SD^3-^. Cell growth was observed 4–10 days after plating. Yeast cells containing *pGBKT7-p53* and *pGADT7-T*, which express *BD* with murine *p53* and *AD* with SV40 large T-antigen, respectively, were used as positive controls. The yeast cell line expressing human *lamin C* with *BD* fusion protein was used as a negative control.

### Accession Numbers

Sequence data and mutant information from this article can be found in the Arabidopsis Genome Initiative or GenBank/EMBL databases under the following accession numbers; AT5G62000 (*ARF2*), AT5G54510 (*GH3.6*), AT1G12560 (*EXPA7*), AT1G27740 (*RSL4*), AT1G15750 (*TPL*), AT1G80490 (*TRP1*), AT3G16830 (*TPR2*), AT5G27030 (*TPR3*), AT3G15880 (*TPR4*), CS24600 (*arf2-6*), and CS24601 (*arf2-7*).

## Results and Discussion

### ARF2 Includes Two Putative TPL/TPR-Binding Motifs in the Middle Domain

Putative EAR and RLFGV motifs in ARFs were previously predicted (Lokerse and Weijers, 2009). Here, we have schematically depicted the location of these motifs in the major ARF domains (Supplementary Figure S1). Only one (ARF19) out of 5 aARFs has the EAR motif in the MD, whereas 13 out of 17 rARFs include one or both of the two motifs. In rARFs, if the RLFGV motif is present it is located in the MD, but, apart from ARF2, the EAR motif resides in either the DBD or PB1 domain.

There are two major reasons for choosing ARF2 to study the role of TPL/TPR-interacting rARF motifs. One is that ARF2 is the only rARF that includes both EAR and RLFGV (RLFGI in ARF2) motifs in the repression MD (Supplementary Figure S1). Motifs located in the MD are more likely to possess repressive activity, that is, the interaction with TPL/TPRs. The other reason for choosing ARF2 is that its defect shows apparent phenotypic effects (Smyth et al., 1990; Ellis et al., 2005; Okushima et al., 2005a,b; Schruff et al., 2006), whereas mutations of most rARFs do not, which allows biological assessment of these putative repressive motifs.

### rARFs Exhibit Repression Activities in the Auxin-Responsive Root Hair Single Cell System

Tissue- or organ-level phenotypes for a gene could derive from complicated interactions among different cell types and organs, which makes it difficult to assess the activation or repression role of a molecular modulator. A single-cell assay system should relieve much of these concerns. In this study, we adopted the root hair system for single-cell-level analysis of the repressive ARF2 motifs. A root hair is the protrusion of a root epidermal cell and serves as a successful single-cell model system to assess auxin transport and signaling because root hair growth is proportional to the cellular auxin level and thus to positive signaling activity (Lee and Cho, 2006; Cho et al., 2007; Li et al., 2009; Ganguly et al., 2010; Lee et al., 2016).

**FIGURE 1:**
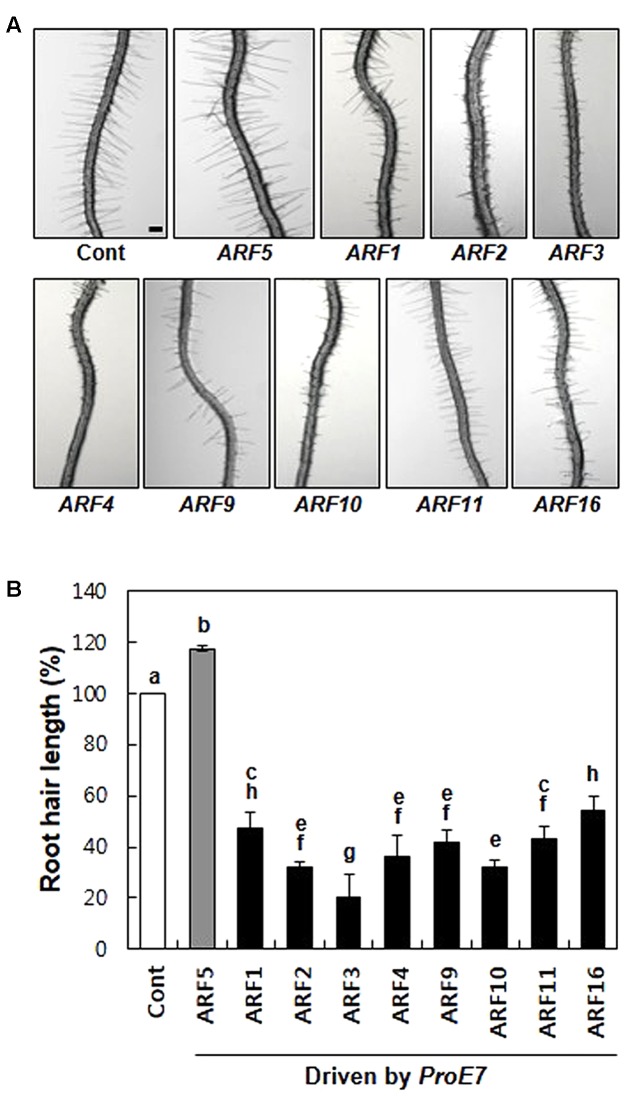
Repressor ARFs inhibit root hair growth. **(A)** Root hair phenotypes of control (Cont; *ProE7:YFP*) and root hair-specific *ARF*-overexpressing lines (*ProE7:ARFs*) in the wild-type background. Bar is 100 μm for all. **(B)** Root hair length of control and *ARF*-overexpressing lines. Error bars indicate ± SE (*n* = 163–1,482 root hairs from 12 to 78 plants from 4 to 6 independent lines for transformants). Statistically significant differences are denoted by different letters (one-way ANOVA with Tukey’s unequal N-HSD *post hoc* test, *P* < 0.05).

First, we tested whether rARFs work as general repressors in root hair growth. We chose eight rARFs (ARF1–4, ARF9–11, and ARF16) and root hair-specifically overexpressed them using the *EXPANSIN A7* promoter (*ProE7*; Cho and Cosgrove, 2002; Kim et al., 2006). All eight rARFs considerably suppressed root hair growth, showing 46–80% inhibition compared with the control level (**Figure 1**), suggesting that these rARFs indeed act as repressors of auxin signaling in the root hair cell. This result is consistent with the previous result that root hair-specific overexpression of aARFs (ARF5, ARF7, and ARF8) enhanced root hair growth (**Figure 1**; Mangano et al., 2017). Taken together, these results demonstrate that the root hair assay system reflects the auxin-signaling directions (positive/negative) of ARFs, which were shown by their transcriptional activities (Ulmasov et al., 1999; Tiwari et al., 2003).

### Mutations of the Putative TPL/TPR-Binding Motifs Suppress ARF2-Mediated Inhibition of Root Hair Growth and *RSL4* Expression

**FIGURE 2:**
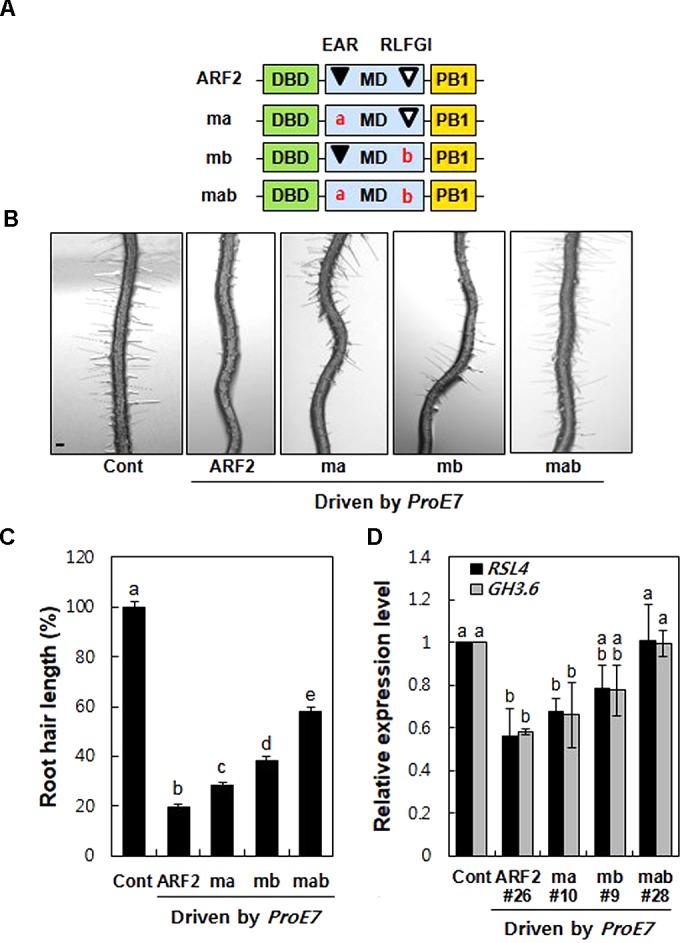
Mutations of the TPL-binding motifs suppress ARF2-mediated inhibition of root hair growth and auxin-responsive root hair gene expression. **(A)** Schematic structures of wild-type (ARF2) and mutant (ma, mb, and mab) ARF2, where ma includes L to R substitutions in the EAR motif (LxLxL), mb includes LF to SS substitution in the RLFGI motif, and mab includes mutations in both EAR and RLFGI motifs. **(B)** Root hair phenotypes of the control (Cont, *ProE7:GFP*) and overexpressing lines for wild-type or mutant *ARF2* genes (ma, mb, and mab) under the *EXPA7* promoter (*ProE7*) in the wild-type background. Bar is 100 μm for all. **(C)** Root hair length of Cont and overexpressing lines of wild-type and mutant *ARF2* genes under *ProE7*. The root hair length is relative to the control value. Error bars indicate ± SE (*n* = 597–3,318 root hairs from 42 to 225 plants from eight independent lines for transformants). **(D)** Transcript levels of *RSL4* and *GH3.6* of Cont and single overexpressing lines for wild-type or mutant *ARF2* genes. Data represent means ± SD from three biological replicates. Statistically significant differences are denoted with different letters (one-way ANOVA with Tukey’s unequal N-HSD *post hoc* test, *P* < 0.05, **C**,**D**).

To assess the repression activity of EAR and RLFGI motifs in ARF2, we generated three mutant forms of ARF2; L-to-R mutation in the EAR motif (ma), L and F-to-S mutation in the RLFGI motif (mb), and both mutations in the EAR and RLFGI motifs (mab) (**Figure 2A**). Wild-type and the three mutant versions of *ARF2* were root hair-specifically overexpressed by *ProE7*, and root hair length was estimated in the transgenic lines.

Overexpression of wild-type *ARF2* inhibited root hair growth by 58–96% in independent transgenic lines (average, ∼80%) compared with the control level (**Figures 2B,C** and Supplementary Figure S3). Consistent with this, the loss-of-function *arf2* mutants grew longer root hairs than the wild type seedling (Supplementary Figure S4). The EAR motif mutation (ma) showed 55–98% root hair growth inhibition depending on transgenic line, averaging 72% inhibition from eight lines. The RLFGI mutation (mb) showed a more suppressive effect on ARF2-mediated root hair inhibition; 50–88% for individual lines and 65% on average. Contrastingly, mutations in both EAR and RLFGI (mab) resulted in much less root hair inhibition, 7–82% for individual lines and an average of 42%. In another set of experiments where we chose the transgenic lines with similar *ARF2* expression levels (Supplementary Figure S5A), a similar pattern was obtained except that both ma and mb effects on root hair inhibition were similar (Supplementary Figure S5B), indicating some probable effects of *ARF2* expression levels on the results between ma and mb in **Figure 2C**. In contrast, mab revealed a much greater suppression effect on ARF2-mediated root hair inhibition (leaving ∼13% root hair inhibition) when similar *ARF2*-expressing lines were compared (Supplementary Figure S5B). Overall, the mutation of both EAR and RLFGI motifs (mab) exhibited a much greater effect on restoration of root hair growth than did the mutation of individual motifs (ma and mb). These results indicate that EAR and RLFGI motifs work cooperatively for the repressive function of ARF2 in the root hair cell.

ROOT HAIR DEFECTIVE SIX-LIKE4 (RSL4), a basic helix-loop-helix transcription factor, is the master modulator of root hair growth. RSL4 regulates a variety of genes involved in root hair growth and morphogenesis (Won et al., 2009; Yi et al., 2010; Vijayakumar et al., 2016). Recently, we have shown that RSL4 directly binds to the root hair-specific *cis*-element (RHE) of *ROOT HAIR SPECIFIC* (*RHS*) genes (Hwang et al., 2017). Most genes (up to 124 genes) regulated by RSL4 include RHE in their promoter regions and encode various worker proteins that play a role in root hair growth and morphogenesis (Hwang et al., 2017). Auxin has long been implicated in root hair growth despite its mechanism of action being unknown (Lee and Cho, 2009). Our recent study demonstrated that aARFs directly bind to AuxRE on the *RSL4* promoter and up-regulate its expression, suggesting that the auxin signaling pathway affects root hair growth by directly targeting *RSL4* (Mangano et al., 2017). Here, because ARF2 shows a repressive role in root hair growth, we tested whether the putative co-repressor-binding motifs of ARF2 are implicated in the repression of *RSL4* expression.

Overexpression of wild-type ARF2 significantly inhibited accumulation of the *RSL4* transcript (**Figure 2D**). While individual mutations of the repressive motifs marginally affected ARF2-mediated inhibition of *RSL4* expression, both mutations almost completely abolished the inhibitory function of ARF2. A similar result was obtained with *GH3.6*, another representative auxin-responsive gene (**Figure 2D**). Consistent with the root hair length result, the transcript analysis suggests that EAR and RLFGI motifs cooperatively operate in ARF2-mediated transcriptional repression of the auxin-responsive genes in the root hair cell.

Overall, our root hair assay system reveals that the EAR and RLFGI motifs of ARF2 play a repressive role during the auxin signaling process in a cell.

### EAR and RLFGI Motifs Are Necessary for ARF2 to Interact With TPL

**FIGURE 3:**
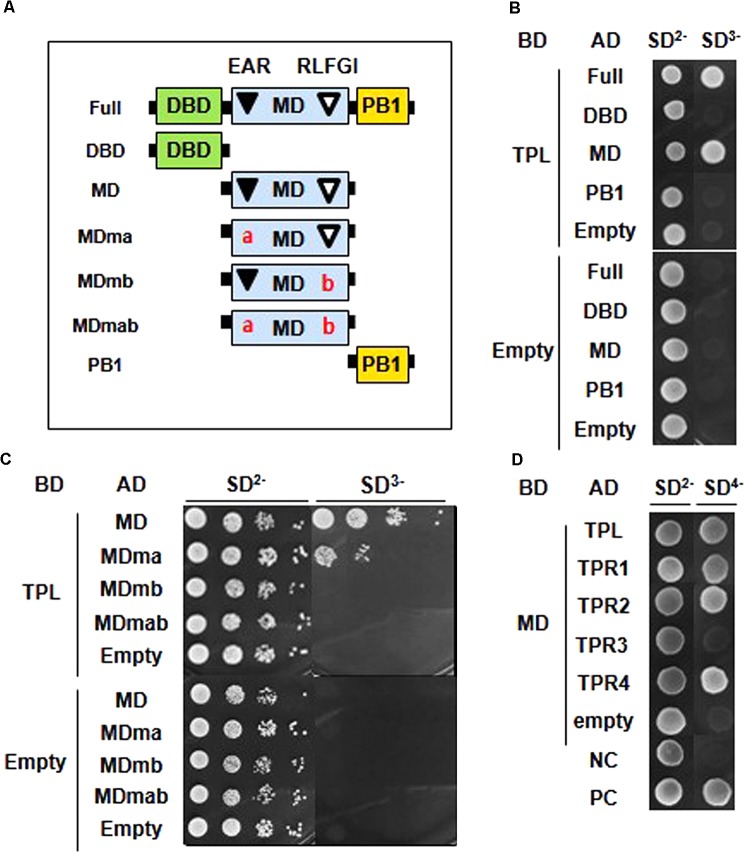
EAR and RLFGI motifs are required for the interaction of ARF2 with TPL/TPRs in the yeast cell. **(A)** Schematic representations of ARF2 full domains (Full), domain deletions (DBD, MD, PB1), and motif-mutated middle domains (MDma, MDmb, and MDmab). **(B)** Yeast two-hybrid assay between TPL and ARF2 domains. TPL is fused with GAL4 DNA binding domain (DB), and full length and domain-deleted ARF2 are fused with GAL4 activation domain (AD). **(C)** Yeast two-hybrid assay between TPL and mutated ARF2-MD series. TPL is fused with GAL4-BD, and ARF2-MD series are fused with GAL4-AD. **(D)** Yeast two-hybrid assay between ARF2-MD (MD) and TPL/TPRs. MD is fused with GAL4-BD, and TPL/TPRs are fused with GAL4-AD (NC, negative control; PC, positive control as described in section “Materials and Methods”). Empty indicates no TPL in the BD-fusion **(B,C)**, and no ARF2 domain **(B)** or no MD **(C)** or no TPL/TPR **(D)** in the AD fusion.

We carried out a Y2H assay to demonstrate whether the putative co-repressor-binding EAR and RLFGI motifs are required for ARF2 to interact with TPL/TPRs. First, we determined which domain of ARF2 is involved in the interaction with TPL. ARF2 full-length, DBD, MD, and PB1 domain were tested in the assay (**Figure 3A**), and the result showed that only the full-length and MD, where EAR and RLFGI motifs are contained, were able to interact with TPL (**Figure 3B**).

Next, MDs with a mutation in EAR (ma), RLFGI (mb), or both motifs (mab) were tested for the interaction with TPL (**Figure 3A**). While the EAR motif mutation moderately disturbed the interaction, the mutation of RLFGI or both motifs completely abolished the interaction between ARF2-MD and TPL (**Figure 3C**), suggesting that RLFGI plays a more critical role in recruiting TPL than EAR. This molecular interaction result is largely reflected in the data showing the role of EAR and RLFGI motifs in ARF2-mediated biological functions in this study.

TPL has four more homologs (TPR1–4) in the Arabidopsis genome. Therefore, we examined whether the EAR and RLFGI-containing ARF2-MD can interact with these TPL homologs. ARF2-MD interacted with TPR1, TPR2, and TPR4 as strongly as it did with TPL but scarcely interacted with TPR3 (**Figure 3D**). This result indirectly suggests that the repressive motifs in the ARF2-MD have a certain level of specificity in choosing TPL homologs.

Our ARF2-TPL/TPR interaction data show a partial consistency with the results from a previous Y2H screening of the whole plant library where ARF2 interacts with TPL, TPR3, and TPR4 but not with TPR1 and TPR2 (Causier et al., 2012a). This previous study also demonstrated that ARF9, containing a RLFGI motif as the only repressive motif (Supplementary Figure S1), interacts with TPL/TPRs. Together with these previous data, our results suggest that RLFGI might be a more preferential motif for rARFs to recruit TPL/TRPs. Two ARFs from a moss, *Physcomitrella patens*, interact with moss TPL homologs (Causier et al., 2012b). These moss ARFs include an EAR (LxLxL) and/or an EAR-like (LxLxPP) motif that could also be a functional repressive motif of moss Aux/IAAs (Paponov et al., 2009). Together with this moss study, our results imply that the rARF EAR motif, which once played a major repressive motif in rARFs, has evolved to work together with the RLFGI motif.

In the interpretation of these molecular interaction data (together with the biological results) of EAR and RLFGI motifs in ARF2, the presupposition is that those motifs should not affect ARF2’s molecular functions other than their interacting function with the co-repressors.

### The Repressive Motifs Are Required for the Native Biological Functions of ARF2

In addition to the root hair system, we tested the function of the two ARF2 repressive motifs in its own expression domain. We expressed wild type (ARF2), EAR mutant (ma), RLFGI mutant (mb), and EAR and RLFGI mutant (mab) by using the *ARF2* promoter in two different loss-of-function *arf2* mutant backgrounds (*arf2-6* and *arf2-7*) and assessed the complementation efficiency of the transgenes.

The *arf2-6* mutant includes the T-DNA in the 12th exon, and the *arf2-7* mutant includes it in the 13th exon (Supplementary Figure S6A). An RT-PCR analysis with two primer sets designed before the 12th exon and after the 13th exon, respectively, indicated that both mutants produce immature *ARF2* transcripts, though the levels could be different between two mutant alleles (Supplementary Figure S6B).

In this study, two native biological functions of ARF2, determination of flowering time and seed size, were analyzed to assess the role of the repressive motifs. ARF2 and ARF3 have shown obvious phenotypic effects in Arabidopsis (Sessions et al., 1997; Nemhauser et al., 2000; Ellis et al., 2005; Okushima et al., 2005a; Schruff et al., 2006; Lim et al., 2010). However, because ARF3 is a truncated ARF without the PB1 domain (Supplementary Figure S1), currently ARF2 is the only canonical rARF that has shown obvious phenotypic effects.

**FIGURE 4:**
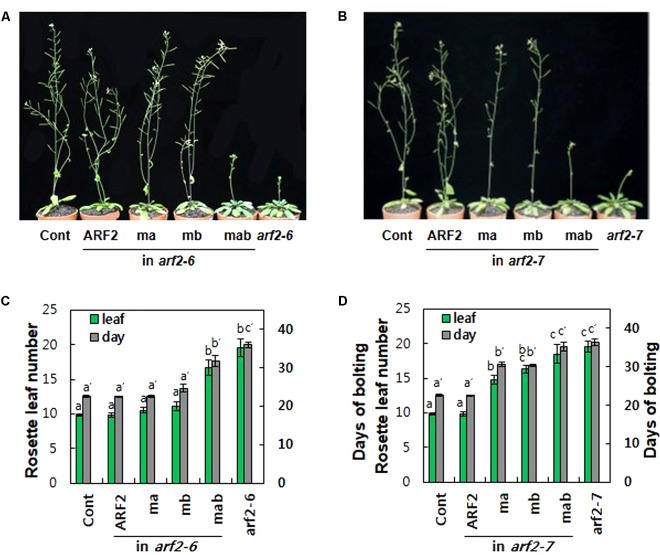
The repressive motifs are needed for ARF2-mediated regulation of flowering time. **(A,B)** Inflorescence phenotypes of 35-day-old wild type (Cont), *arf2* mutants, and *arf2* mutants complemented with wild-type (ARF2) or mutated (ma, mb, and mab) *ARF2* under *ProARF2*. **(C,D)** Flowering time of the lines shown in **A** **(C)** and **B** **(D)** in terms of rosette leaf number at bolting time. Error bars indicate ± SE (*n* = 7–12 plants from 2 to 3 independent lines). Statistically significant differences are denoted with different letters (one-way ANOVA with Tukey’s unequal N-HSD *post hoc* test, *P* < 0.05).

Loss-of-function *arf2* mutants show late flowering phenotypes under long day conditions in Arabidopsis (**Figure 4**; Ellis et al., 2005; Okushima et al., 2005a; Lim et al., 2010). In terms of rosette leaf number at the bolting time, while 9.8 leaves emerged in wild-type plants, 19.6 leaves emerged in both *arf2* mutants (**Figures 4C,D**). The time taken for bolting was 22.6 days for wild type and 36 and 36.4 days for *arf2-6* and *arf2-7* mutants, respectively, demonstrating that the ARF2 defect considerably delayed flowering time. Complementation with wild-type ARF2 completely rescued the delayed flowering time phenotype in both *arf2* mutant backgrounds. Individual mutations of EAR and RLFGI motifs did not affect ARF2-mediated delay of flowering time in the *arf2-6* background but significantly hampered the ARF2 effect in the *arf2-7* background. In contrast, the mutation of both repressive motifs considerably or completely abolished the inhibitory ARF2 function in flowering time in both *arf2* backgrounds. The phenotypic differences of ma and mb between the two *arf2* mutant backgrounds could derive from the different residual functionalities of the two *arf2* mutant proteins with differently truncated forms, as speculated from the result that the two *arf2* mutant genes produced immature transcripts (Supplementary Figure S6B). However, the results from both mutant backgrounds consistently support the idea that EAR and RLFGI motifs cooperatively operate and work as the main repressive motifs for the repressive function of ARF2.

**FIGURE 5:**
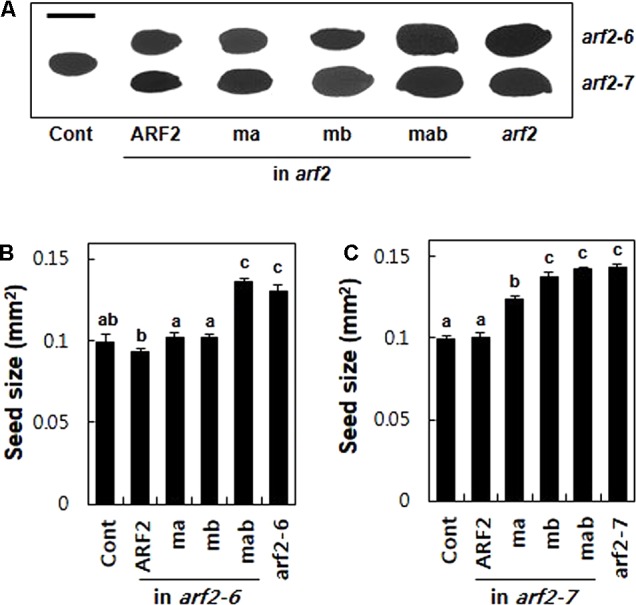
The determination of seed size by ARF2 requires its TPL/TPR-binding motifs. **(A)** Representative seed images of wild type (Cont), *arf2* mutants, and the *arf2* mutants complemented with wild-type (ARF2) or mutated (ma, mb, and mab) *ARF2* under *ProARF2*. Bar is 500 μm. **(B,C)** Quantitative seed size analyses of the lines shown in **A**. Seed size was quantified as the area of the two-dimensional seed image. Data represent means ± SE (*n* = 24–244 seeds from 3 to 7 independent lines for transformants). Statistically significant differences are denoted with different letters (one-way ANOVA with Tukey’s unequal N-HSD *post hoc* test, *P* < 0.05).

To further evaluate the repressive motifs of ARF2, we took advantage of another known *arf2* mutant phenotype, that is, enlarged seed size (Okushima et al., 2005a; Schruff et al., 2006). The *arf2* mutants produced 31 and 44% bigger seeds (as a measure of 2-dimensional seed area) than the wild-type plant for *arf2-6* and *arf2-7*, respectively, suggesting that ARF2 works as a negative regulator in seed growth. The complementation of the mutants with wild-type ARF2 restored the wild-type level seed size (**Figure 5**). While introduction of the mutated ARF in either EAR (ma) or RLFGI (mb) motifs in the *arf2-6* background did not disturb ARF2-mediated inhibition of seed growth, the mutant ARF forms in the *arf2-7* background significantly compromised the inhibitory ARF2 function with a stronger effect by mb than ma (**Figure 5C**). As previously mentioned, this phenotypic difference between *arf2-6* and *arf2-7* backgrounds could be due to their different allelic effects. In contrast, the mutation of both EAR and RLFGI motifs showed similar effects on the seed size in both mutant backgrounds, that is, complete abolishment of the inhibitory ARF2 function. Together with the flowering time data, this result again suggests that EAR and RLFGI motifs are the main repressive motifs that recruit TPL/TPRs in the ARF2 protein and both motifs work together to recruit the co-repressors during ARF2-mediated native biological processes.

In this study, we have demonstrated the molecular interaction between TPL/TPRs and ARF2 EAR/RLFGI motifs and the function of these repressive motifs in ARF2-mediated gene regulation and three biological processes. In terms of molecular interaction, RLFGI was shown to be more critical than EAR for TPL recruitment, whereas this difference is partially reflected in the biological parameters with minor effects. However, because disruption of both EAR and RLFGI motifs almost fully abolishes the ARF2 functions in both the molecular interaction and biological processes, these co-repressor-binding motifs likely play a major role, in a cooperative manner, in the repressive function of ARF2. Further studies should follow to extend this view to other rARFs that include these repressive motifs.

In our root hair assay, all the tested rARFs revealed inhibitory effects on auxin-responsive root hair growth (**Figure 1**). Among these rARFs, ARF2 and ARF11 contain both EAR and RLFGI motifs, ARF1, ARF3, ARF4, and ARF9 contain only a RLFGI motif, ARF10 contains only an EAR-like motif, and ARF16 contains no known repressive motif (Supplementary Figures S1, S2). Since a RLFGI or RLFGI-like motif is present in most rARFs and located in similar MD regions, this could be a general repressive motif among rARFs. Conversely, the EAR or EAR-like motif in rARFs other than ARF2 is located not in the MD but in either the DBD or PB1 domain, leaving uncertainty about their functionality. Although ARF2 requires both EAR and RLFGI motifs for its full repressive function, our root hair assay results open the possibility that a single motif (for example, RLFGI) is enough to execute a full repressive function as shown for ARF3, which contains only a RLFGI motif but is as strong as ARF2 in inhibiting root hair growth (**Figure 1B**). It is intriguing that ARF16, which has no repressive motif, is able to considerably inhibit root hair growth. This suggests that there could be as yet unidentified repressive motifs, or a rARF may repress the target gene by simply competing with aARFs for binding to AuxRE as both ARF types are able to bind to the same AuxRE (Boer et al., 2014).

## Author Contributions

H-SC, MS, and H-TC conceived the research plans, designed the experiments, and analyzed the data. H-SC performed most of the experiments. MS contributed to the yeast two-hybrid analysis. H-SC and H-TC wrote the article.

## Conflict of Interest Statement

The authors declare that the research was conducted in the absence of any commercial or financial relationships that could be construed as a potential conflict of interest.
