# Supplementary material for: Two TPL-Binding Motifs of ARF2 Are Involved in Repression of Auxin Responses
Source: Front Plant Sci. 2018 Mar 21;9:372. doi: 10.3389/fpls.2018.00372 (PMC5871684; doi:10.3389/fpls.2018.00372)
Supplement: Supplementary file 1 [file Table_1.PDF]

TABLE S1. Primer list

Subject			Primer name	Primer sequence (5' to 3')
Transgene Construct	ARF1		ARF1_PcF	GTTTTTAATTAATAGAGGAGGATAGGTCC
			ARF1_AsR	TGCTGGCGCGCCCATATTTCCTACGTGGC
	ARF2	ARF2	ARF2_DBF	ATAGTCGACTAATGGCGAGTTCGGAG
			ARF2_BmR	ATAGGATCCTTAAGAGTTCCCAGCGCT
		ARF2ma	ARF2_DBF	ATAGTCGACTAATGGCGAGTTCGGAG
			Mega primer1-maF	CACTCTGGTGCCTCCGCGAAGGCACATGAATCT
			Mega primer1-R	ATAGGATCCTTAAGAGTTCCCAGCGCT
		ARF2mb	ARF2_DBF	ATAGTCGACTAATGGCGAGTTCGGAG
			Mega primer1-mbF	GAACTGCAGGTCCTCTGGCATT
			Mega primer1-R	ATAGGATCCTTAAGAGTTCCCAGCGCT
		ARF2mab	ARF2_DBF	ATAGTCGACTAATGGCGAGTTCGGAG
			Mega primer2-maF	CACTCTGGTGCCTCCGCGAAGGCACATGAATCT
			Mega primer1-mbF	GAACTGCAGGTCCTCTGGCATT
			Mega primer1-MD-R	GCAGTCGACACTCCTACTTGAGTTGGT
			Mega primer3-R	ATAGGATCCTTAAGAGTTCCCAGCGCT
	ARF3		ARF3_PcF	GCTCTTAATTAAATCTCTGTTTCTCTCTC
			ARF3-AsR	AAGCGGCGCGCCAATAAAACACAGAAACC
	ARF4		ARF4-PcF	GCTCTTAATTAAGAAGCTTTTTCAATGGA
			ARF4-AsR	CTTTGGCGCGCCAAACACACTTTAAAACA
	ARF9		ARF9_PcF	AGAGTTAATTAATGTTTTGGTGTGACTGAT
			ARF9_AsR	GGATGGCGCGCCTTGTTTAAGCCATGCGC
	ARF10		ARF10_PcF	CAACTTAATTAAGATGGAGCAAGAGAAAA
			ARF10_AsR	AAAGGGCGCGCCTTATTCCTCAATATGCT
	ARF11		ARF11_PcF	ATTTTTAATTAAAATCATGAGCCAAACAAGCTTAG
			ARF11_AsR	AATTGGCGCGCCTTGAGAGAGTCTCCAAG
	ARF16		ARF16_PcF	TTTCTTAATTAACGGTCACAAAAAATATG
			ARF16_AsR	CTTCGGCGCGCCAACAAAACACTCTGCCA
	ProARF2		pARF2_H3F	ATCAAGCTTACACAAGAAAATAGAAGAG
			pARF2_SlR2	ATCGTCGACACCTTCCGAAGCTCAGATCT

(continued)

TABLE S1. **Primer list** (continued)

Subject		Primer name	Primer sequence (5’ to 3’)
Y2H assay constructs	TPL	TPL_SlF	GGAGTCGACAGAAAACATGTCTTCTCTTA
		TPL_XmR	TATCCCGGGTCATCTCTGAGGCTGATCAGATG
	TPR1	TPR1_SlF	CTTGTCGACACTGAGTGGCAAAATCAATC
		TPR1_SlR	CACTGCAGATCATCTCTGAGGCTGGTC
	TPR2	TPR2_SmF	TAGCCCGGGATGTCGTCTTTGAGCAGAG
		TPR2_SmR	TCACCCGGGTGTCAACTAACTTAACTGACAT
	TPR3	TPR3_SmF	AGACCCGGGGAGAATGTCGTCGTTGAGTC
		TPR3_SmR	ATCGCCCGGGCTGGTTTGTTCATCTTTGTAA
	TPR4	TPR4_BmF	ACGAGGATCCGAGGATATGTCGTCACTCAG
		TPR4_BmR	ATCAGGATCCCTGCTTCCATCTCCAACTAC
	ARF2 full	ARF2_DBF	ATAGTCGACTAATGGCGAGTTCGGAG
		ARF2_BmR	ATAGGATCCTTAAGAGTTCCCAGCGCT
	DBD	ARF2_DBD_F	ATAGTCGACTAATGGCGAGTTCGGAG
		ARF2_DBD_R	ATAGTCGACTGGCTCTACTTTCCACGG
	MD	MD_SmF	GCAGTCGACTAATGAGATTTGAAGGC
		MD_R	GCAGTCGACACTCCTACTTGAGTTGGT
	MDma	MD_SmF	GCAGTCGACTAATGAGATTTGAAGGC
		Mega primer1-maF	CACTCTGGTGCCTCCGCGAAGGCACATGAATCT
		Mega primer1-MD-R	GCAGTCGACACTCCTACTTGAGTTGGT
	MDmb	MD_SmF	GCAGTCGACTAATGAGATTTGAAGGC
		Mega primer1-mbF	GAACTGCAGGTCCTCTGGCATT
		Mega primer1-MD-R	GCAGTCGACACTCCTACTTGAGTTGGT
	MDmab	MD_SmF	GCAGTCGACTAATGAGATTTGAAGGC
		Mega primer2-maF	CACTCTGGTGCCTCCGCGAAGGCACATGAATCT
		Mega primer1-mbF	GAACTGCAGGTCCTCTGGCATT
		Mega primer1-MD-R	GCAGTCGACACTCCTACTTGAGTTGGT
	PB1	ARF2_PB1_F	ATAGTCGACTAATGAATGGGACAGACTCA
		ARF2_PB1_R	ATAGTCGACTTAAGAGTTCCCAGCGCT
QRT-PCR	RSL4	RSL4_qF842	GTGCCAAACGGGACAAAAGT
		RSL4_qR1097	TTGTGATGGAACCCCATGTC
	ARF2	ARF2_qF3471	TGGAGAGTTGATGGCTCCTA
		ARF2_qR3735	CTCGCTCCTACAGCTTAAAGTC
	ACT7	ACT7-rt-F	TCCCTCAGCACCTTCCAACAG
		ACT7-rt-R	CAATTCCCATCTCAACTAGGG
	GH3.6	GH3.6-rt-F	GGACTTTCGATAAGCTCATGGATTATGCG
		GH3.6-rt-R	GTTTCCTCTTTAGTTACTCCCCCATTGC
