# Supplementary figures and images for: Two TPL-Binding Motifs of ARF2 Are Involved in Repression of Auxin Responses
Source: Front Plant Sci. 2018 Mar 21;9:372. doi: 10.3389/fpls.2018.00372 (PMC5871684; doi:10.3389/fpls.2018.00372)

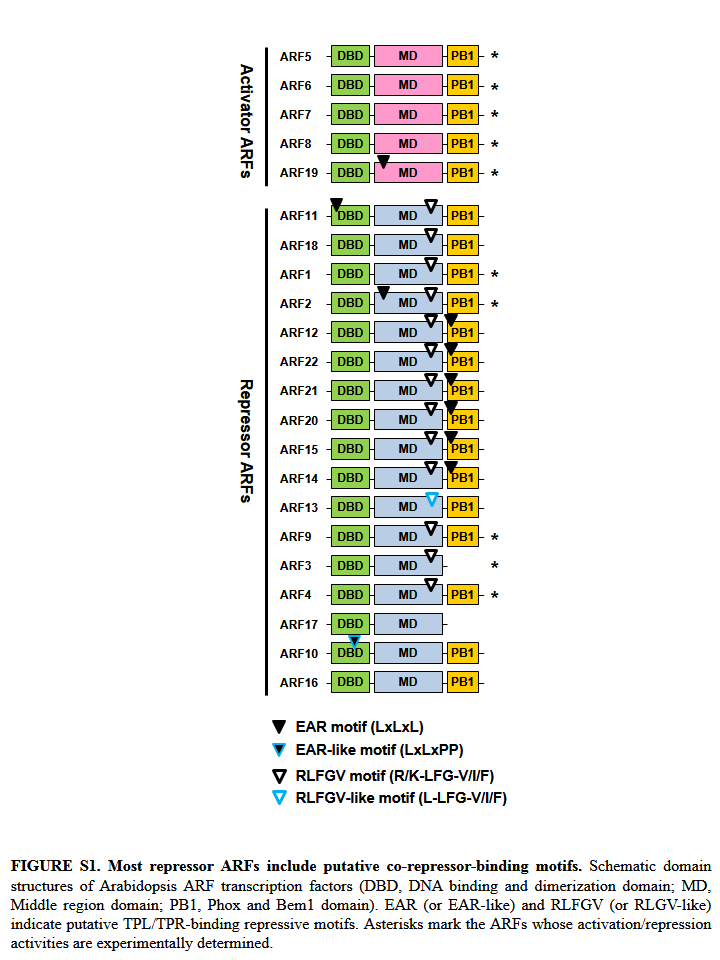

Supplement: Supplementary file 2 [file Image_1.TIF]

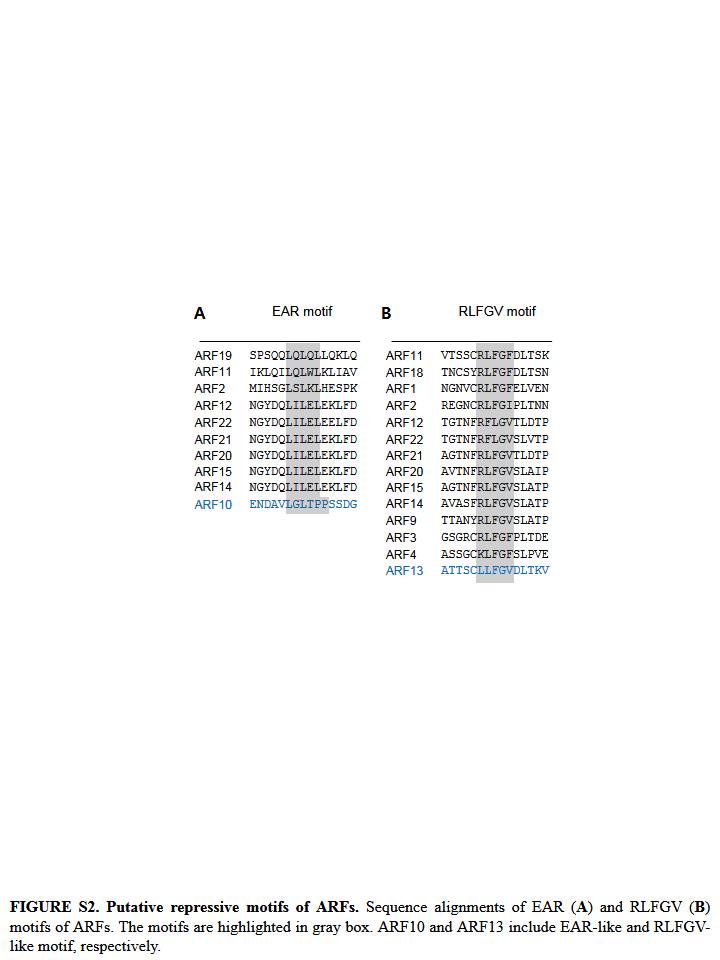

Supplement: Supplementary file 3 [file Image_2.TIF]

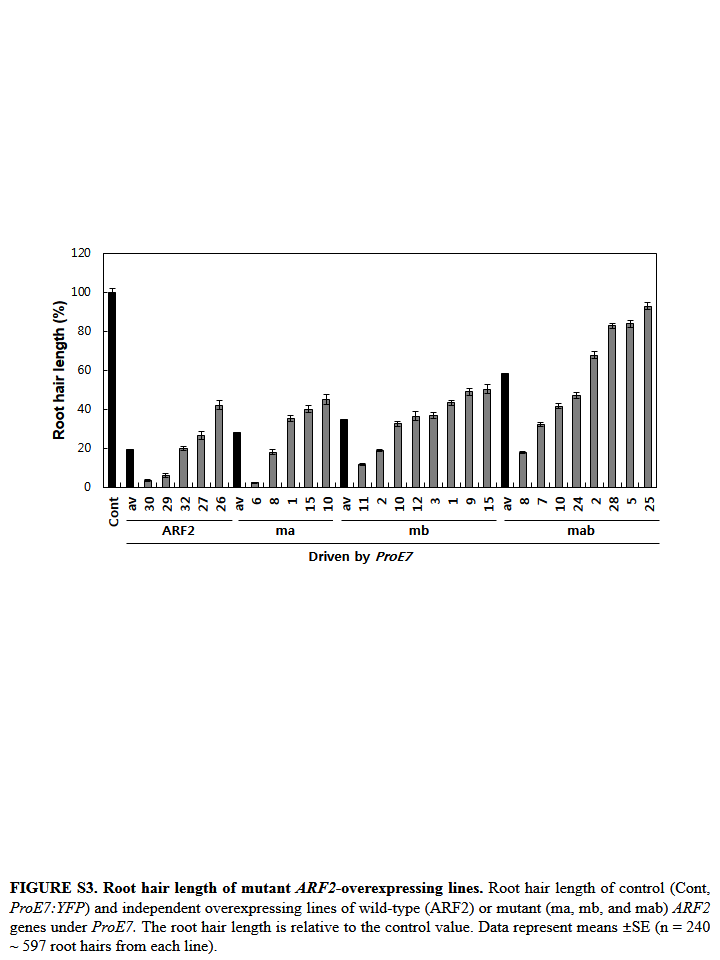

Supplement: Supplementary file 4 [file Image_3.TIF]

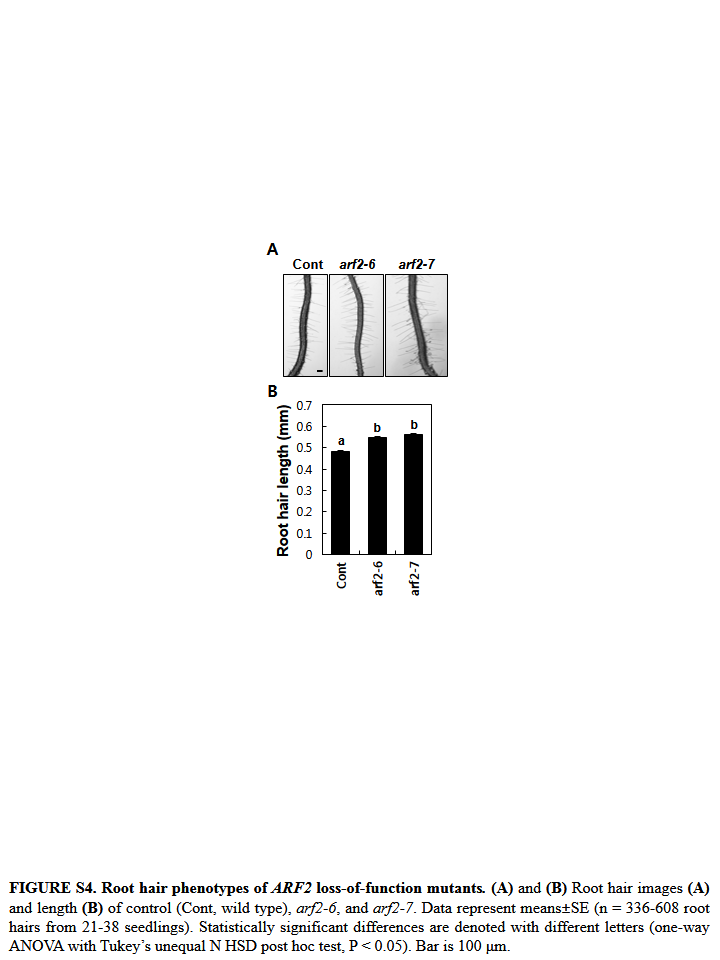

Supplement: Supplementary file 5 [file Image_4.TIF]

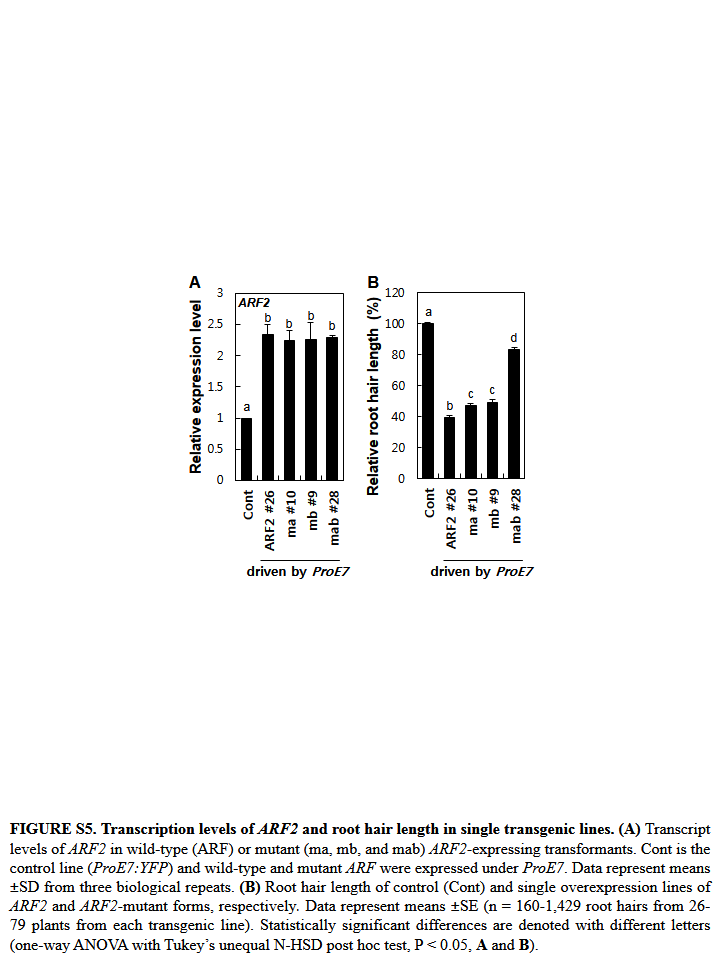

Supplement: Supplementary file 6 [file Image_5.TIF]

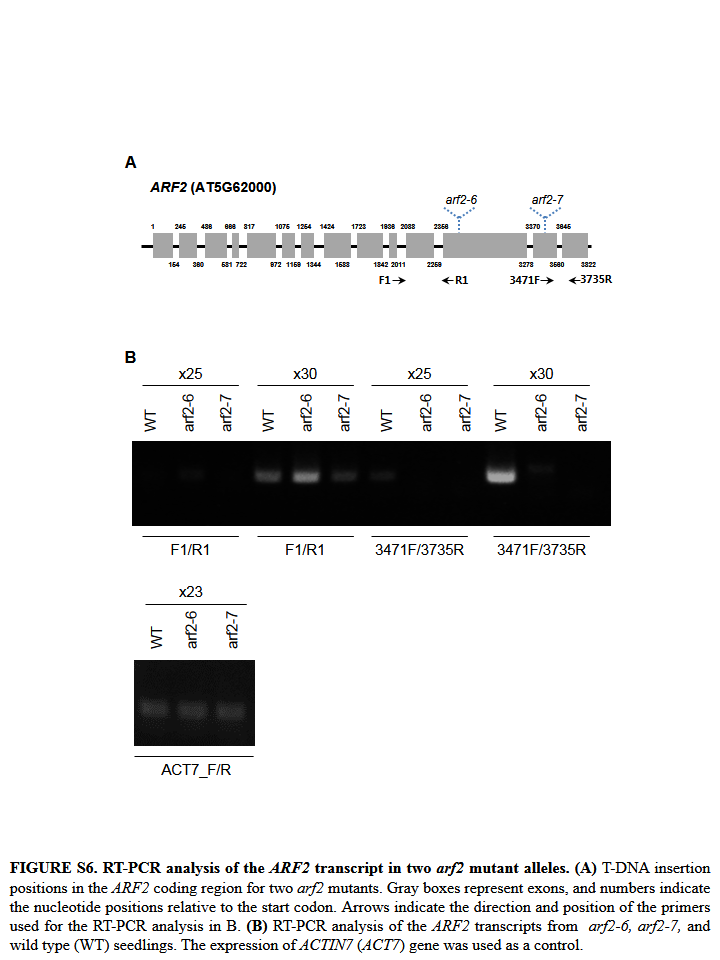

Supplement: Supplementary file 7 [file Image_6.TIF]
